# Histological transformation to signet-ring cell carcinoma in a patient with clinically aggressive poorly differentiated adenocarcinoma of the ascending colon after response to chemotherapy plus cetuximab: a case report

**DOI:** 10.1186/s12957-023-03053-2

**Published:** 2023-06-07

**Authors:** Hideki Nagano, Shigekazu Ohyama, Atsushi Sato, Jun Igarashi, Tomoko Yamamoto, Masumi Kadoya, Mikiko Kobayashi

**Affiliations:** 1 Department of Surgery, Marunouchi Hospital, 1-7-45, Nagisa Matsumoto, Nagano, 390-0841 Japan; 2 Department of Radiology, Marunouchi Hospital, 1-7-45, Nagisa Matsumoto, Nagano, 390-0841 Japan; 3 Department of Pathology, Marunouchi Hospital, 1-7-45, Nagisa Matsumoto, Nagano, 390-0841 Japan

**Keywords:** Histological transformation, Poorly differentiated adenocarcinoma, Signet-ring cell carcinoma, Ascending colon cancer, Chemotherapy, Cetuximab

## Abstract

**Background:**

Alteration of chemosensitivity or tumor aggressiveness in response to chemotherapy has been reported, and liquid biopsy assessment during chemotherapy for colorectal cancers has confirmed the acquisition of mutations in various oncogenes. However, the occurrence of histological transformation seems to be extremely rare in colorectal cancers, and the few existing case reports of this transformation are from lung cancer and breast cancer. In this report, we describe the histological transformation of clinically aggressive scirrhous-type poorly differentiated adenocarcinoma of the ascending colon to signet-ring cell carcinoma in almost all recurrent tumors that were confirmed by autopsy after response to chemotherapy plus cetuximab.

**Case presentation:**

A 59-year-old woman visited our hospital with whole abdominal pain and body weight loss and was diagnosed with scirrhous-type poorly differentiated adenocarcinoma of the ascending colon with aggressive lymph node metastases. The intrinsic chemosensitivity of the tumors was evident upon initiation of mFOLFOX6 plus cetuximab therapy, and right hemicolectomy was performed, and the tumor obviously remained in the peripancreatic area, paraaortic region, or other retroperitoneal areas. The ascending colon tumors mainly consisted of poorly differentiated adenocarcinoma and were not associated with signet-ring cell components except for minute clusters in a few lymphatic emboli in the main tumor. Chemotherapy was continued, and metastases were eliminated at 8 months after the operation; this response was maintained for an additional 4 months. Discontinuation of chemotherapy plus cetuximab resulted in immediate tumor recurrence and rapid expansion, and the patient died of the recurrent tumor 1 year and 2 months after the operation. Autopsy specimens revealed that almost all of the recurrent tumors exhibited transformation and consisted of signet-ring cell histology.

**Conclusion:**

This case might suggest that various oncogene mutations or epigenetic changes resulting from chemotherapy, especially regimens that include cetuximab, contribute to the transformation of non-signet-ring cell colorectal carcinoma to signet-ring cell carcinoma histology and can promote the aggressive clinical progression characteristic of signet-ring cell carcinoma.

## Background

Advances in chemotherapy, including anti-epidermal growth factor receptor (EGFR) tyrosine kinase inhibitors (TKIs), anti-vascular endothelial growth factor (VEGF) TKIs, inhibitors of v-raf murine sarcoma viral oncogene homolog B1 (BRAF) V600 kinase, or immune checkpoint inhibitors (ICIs) for metastatic colorectal cancers (CRCs), have provided benefits such as improvement in overall survival (OS) or progression-free survival (PFS) of patients over the past decade; however, there are still many cancers that are unresponsive or develop chemoresistance [1]. Liquid biopsy assessment during chemotherapy for CRCs has proved the acquisition of mutations in various oncogenes, including RAS or BRAF genes, tumor suppressor genes, or genes involved in the deoxyribonucleic acid (DNA) repair process, and the development of chemotherapy resistance [2–4]; the acquisition of these mutations has been shown to occur even in chemosensitive tumors to some degree [5]. Alteration of chemosensitivity or tumor aggressiveness in response to chemotherapy has been reported; however, to the best of our knowledge, the occurrence of histological transformation seems to be extremely rare for CRCs, and the few existing case reports were from lung cancer and breast cancer [6–9].

Signet-ring cell carcinoma (SRCC) is a rare histological type of CRC with an approximate incidence of 1%; SRCC is diagnosed when > 50% of tumor cells have prominent intracytoplasmic mucin, typically with displacement and molding of the nucleus [10]. It is associated with a scirrhous appearance, aggressive manifestation including a higher frequency of locoregional lymph node involvement, lympho-vascular invasion and perineural infiltration, peritoneal dissemination, and adverse prognosis [11–14].

In this report, we describe the histological transformation of clinically aggressive scirrhous-type poorly differentiated adenocarcinoma (PDA) of the ascending colon to SRCC in almost all recurrent tumors that were confirmed by autopsy after response to chemotherapy plus cetuximab.

## Case presentation

**Fig. 1:**
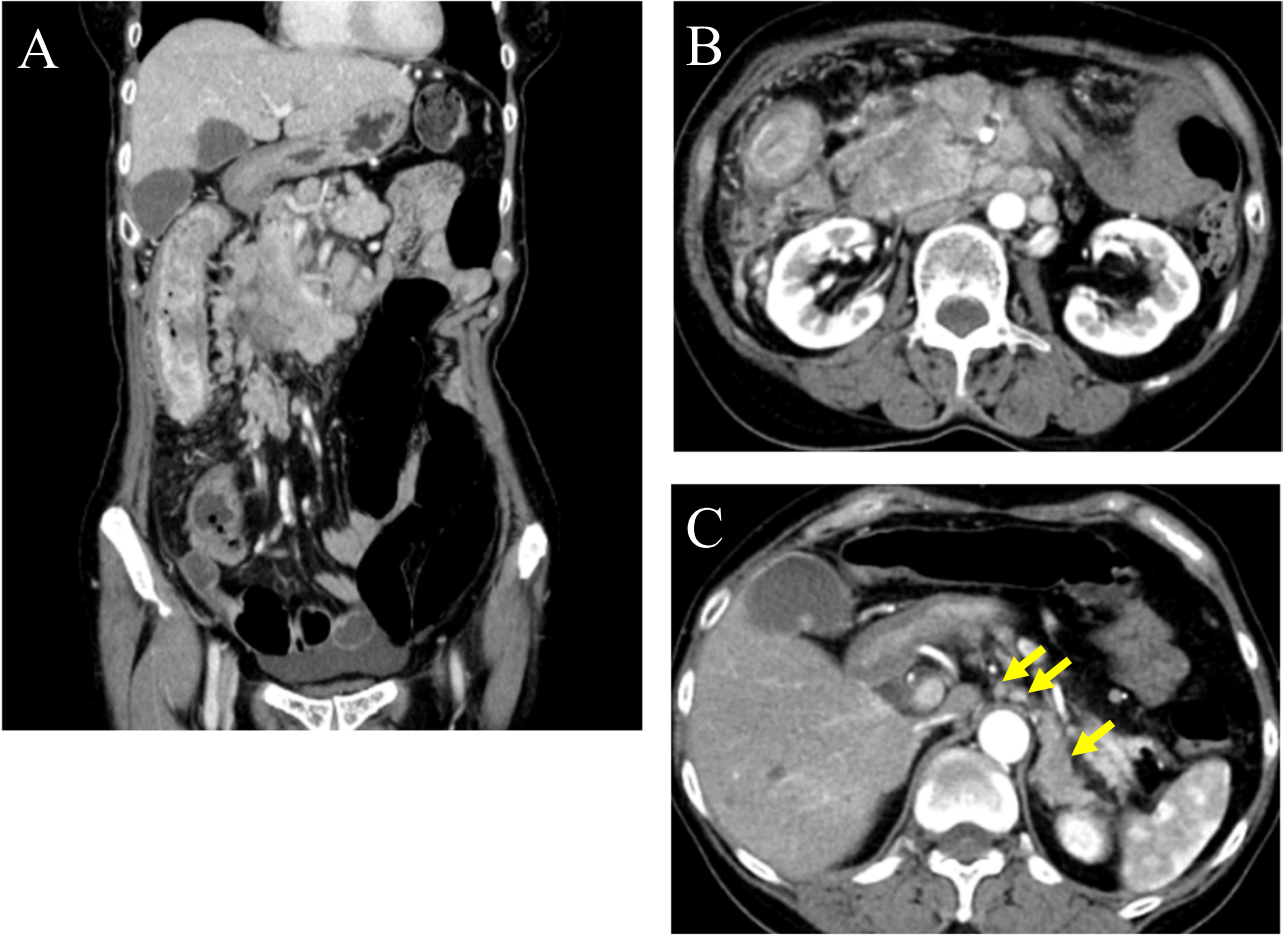
Contrast-enhanced CT findings at diagnosis. **A** CT showed wall thickness of the whole length of the ascending colon and aggressive metastasis to locoregional lymph nodes and peripancreatic lymph nodes. **B** Paraaortic lymph node metastases and an enlarged pancreatic head are shown. **C** Lymph node metastases of the peri-left adrenal region (arrows)

**Fig. 2:**
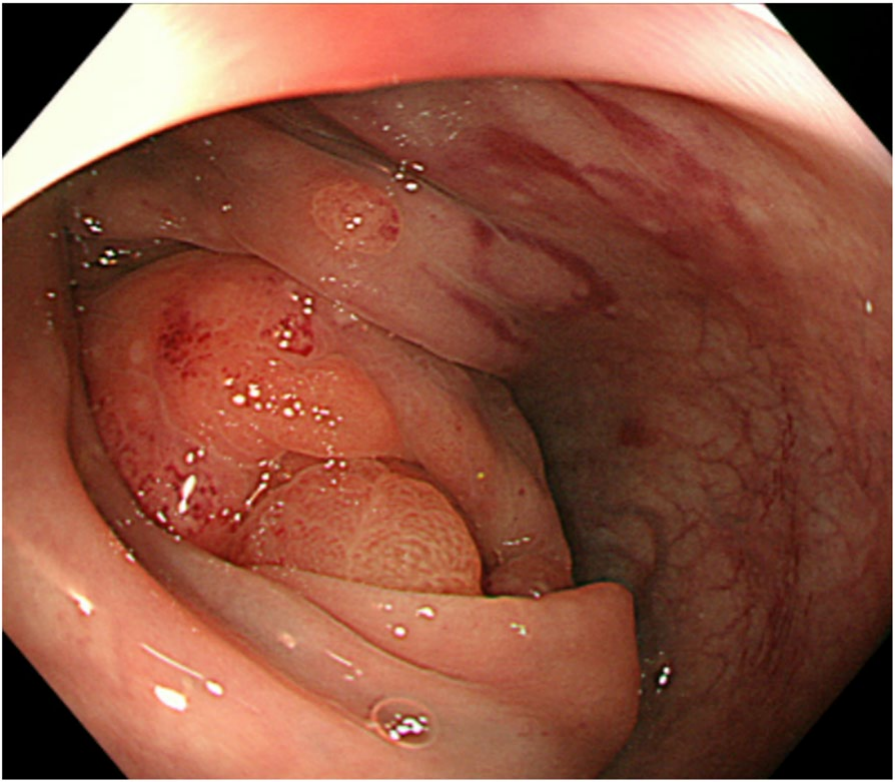
Colonoscopy findings. Colonoscopy revealed the anal side margin of the circumferential tumor on the hepatic flexure. Biopsy was taken from the tumor, and the tumor was diagnosed as PDA of the ascending colon using immunostaining to discriminate it from malignant lymphoma

**Fig. 3:**
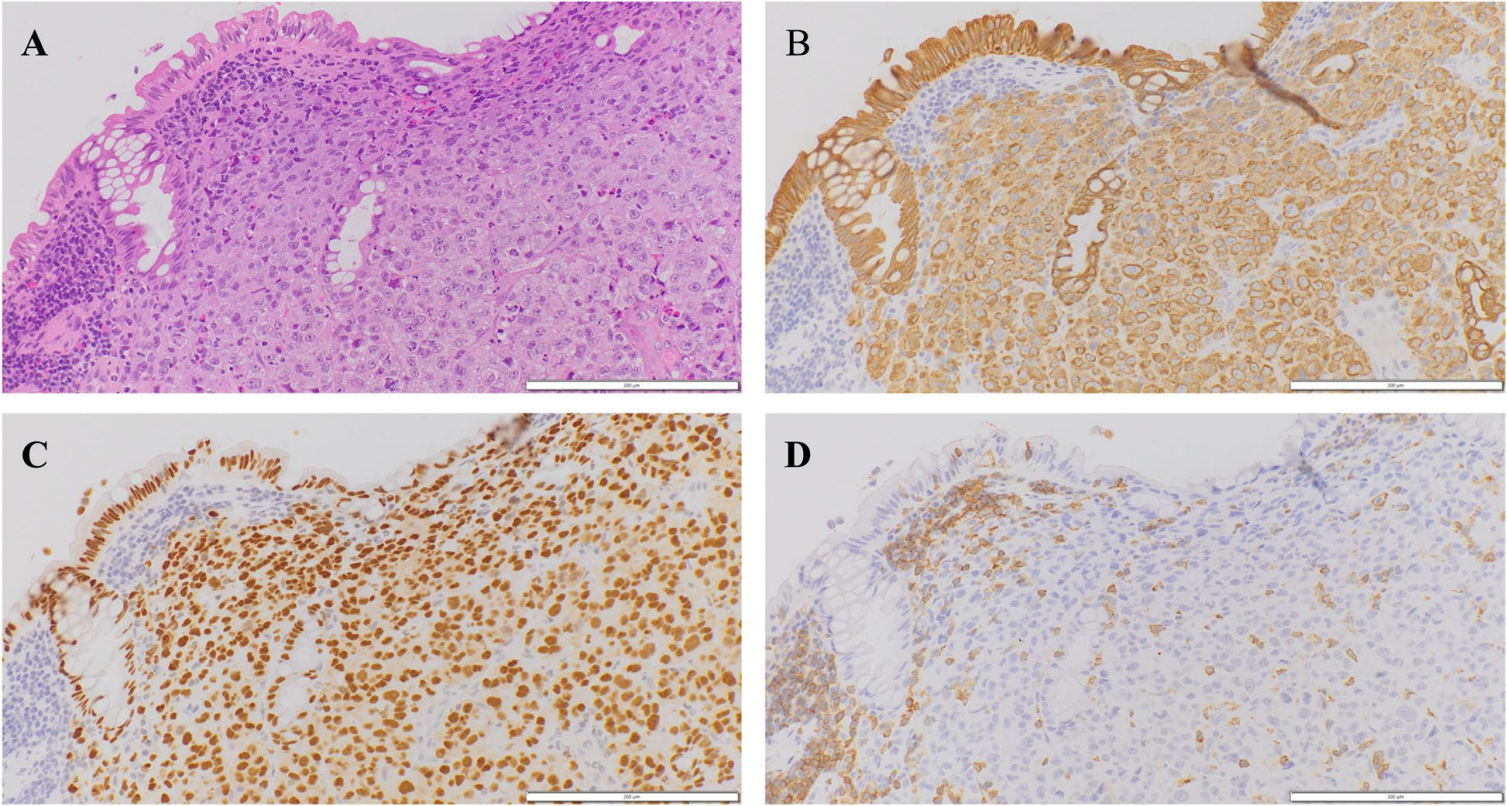
Histopathology and immunostaining of ascending colon biopsy specimens. Hematoxylin and eosin staining (**A**) and immunostaining for CK AE1/AE3 (**B**), CDX2 (**C**), and CD45 (**D**). Tumor cells show poorly differentiated adenocarcinoma-like findings (**A**). These cells are positive for CK AE1/AE3 (**B**) and CDX2 (**C**) and negative for CD45 (**D**). **A**–**D** Original magnification × 200. Each bar displayed at the bottom right indicates 200 μm

**Fig. 4:**
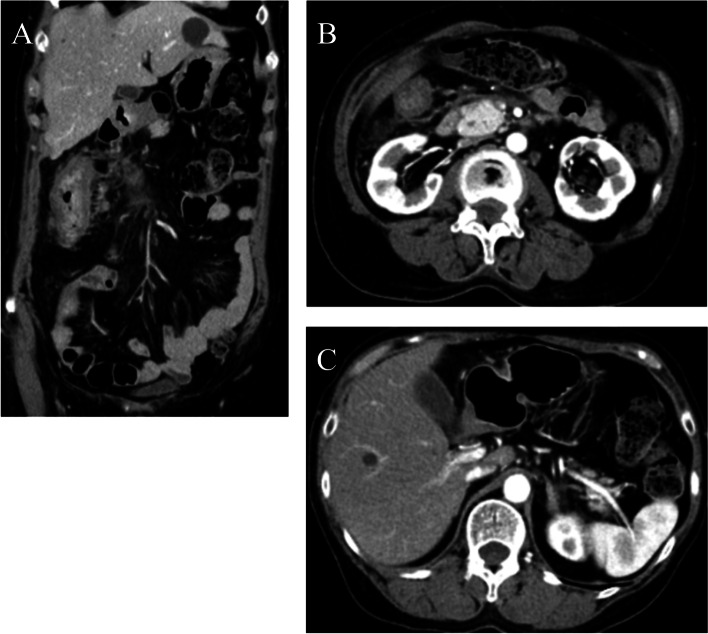
Contrast-enhanced CT findings after 6 courses of chemotherapy. **A** Although the wall thickness of the ascending colon remained consistent, the enhancement of the colon wall was decreased, as was the enhancement of lymph nodes near the tumor. **B** Paraaortic lymph nodes and enlargement of the pancreas head disappeared. **C** Lymph node metastases in the peri-left adrenal region were also diminished

A 59-year-old female presented with whole abdominal pain and body weight loss of 4 kg and visited our hospital in September 2020. Computed tomography (CT) revealed wall thickening of the whole length of the ascending colon and aggressive metastasis to locoregional lymph nodes and those located around the pancreas head and body, paraaortic region, celiac artery, right prerenal space, and peri-left adrenal gland (Fig. 1). Colonoscopy was carried out, and the anal side margin of a circumferential tumor on the hepatic flexure was found; the endoscope was not able to pass through the tumor lesion due to significant narrowing (Fig. 2). A biopsy was taken. Tumor cells showed PDA-like histology with scant intracytoplasmic mucin, resembling the appearance of malignant lymphoma (Fig. 3A). Then, immunohistochemical staining was performed, revealing positive staining for CK AE1/AE3 and CDX2 and negative staining for CD45 (Fig. 3B–D). Thus, a diagnosis of PDA of the ascending colon was made. Genetic mutations in KRAS, NRAS, and BRAF were absent. Serum levels of tumor markers such as CEA and CA19-9 showed normal levels of 2.1 ng/mL and 20.5 U/dL, respectively. Considering the aggressive nature of the colon cancer accompanied by metastasized lymph nodes but no bowel obstruction, chemotherapy, namely, mFOLFOX6, was initially administered followed by 5 courses of mFOLFOX6 plus cetuximab. CT after chemotherapy showed an obviously decreased size of metastasized lymph nodes (Fig. 4). To achieve primary tumor excision to prevent recurrence and subsequent stenosis or ileal stoma construction, right hemicolectomy with palliative lymph node dissection was carried out in February 2021. During the operation, peritoneal or liver metastases were not found. However, around the pancreas, white scarring was significant, and thickening and stiffening were obvious from the root of the mesocolon to the peripancreatic area; furthermore, the mesocolon of the hepatic flexure was unable to be detached from the pancreas. The tumor obviously remained in the peripancreatic area, paraaortic region and other retroperitoneal areas. The patient showed uneventful recovery and was discharged on postoperative day 14.

**Fig. 5:**
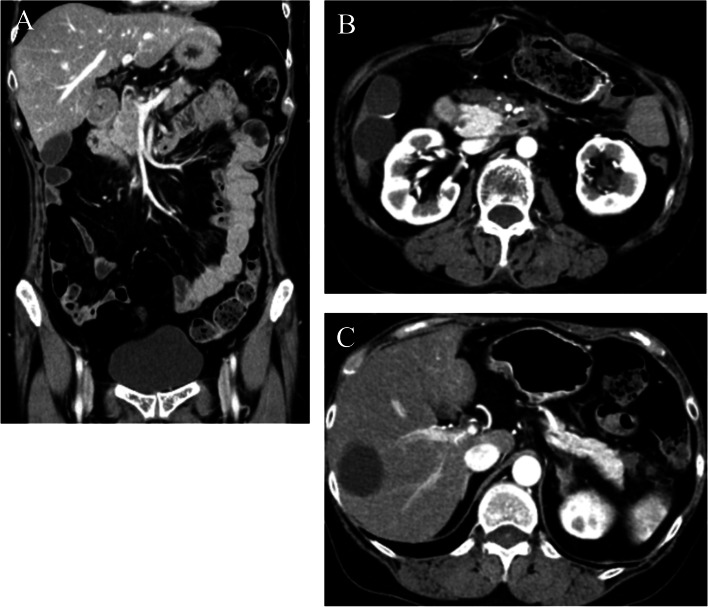
Contrast-enhanced CT findings after adjuvant chemotherapy was continued for 8 months after colectomy. **A** CT showed elimination of peripancreatic lymph node metastases. **B** Elimination of paraaortic lymph node metastases and enlargement of the pancreas head were maintained. **C** Elimination of lymph node metastases of the peri-left adrenal region was also maintained

**Fig. 6:**
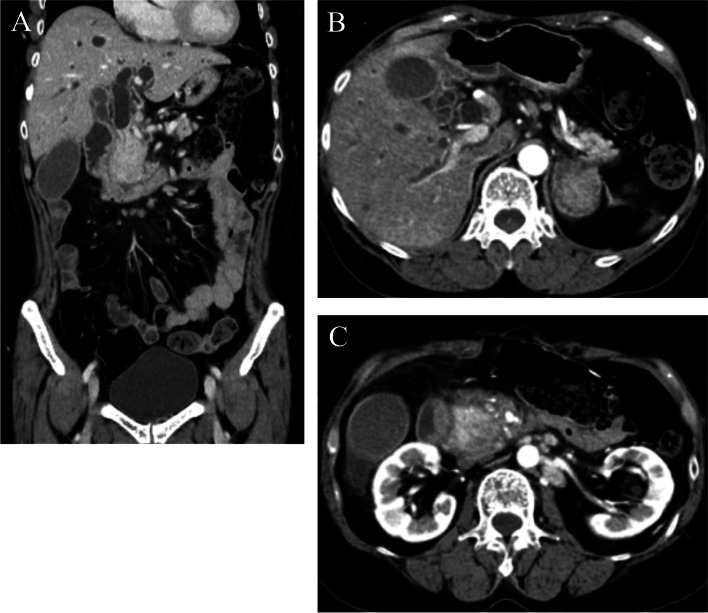
Contrast-enhanced CT one month after confirmation of maintenance and no evidence of recurrence by previous CT. **A** CT revealed choledochal obstruction due to the mass in the pancreas head and poor extension of the duodenum due to tumor invasion from the pancreas. **B** Enlarged metastatic lymph nodes in the peri-left adrenal region are shown. **C** Swelled paraaortic lymph nodes are also revealed

**Fig. 7:**
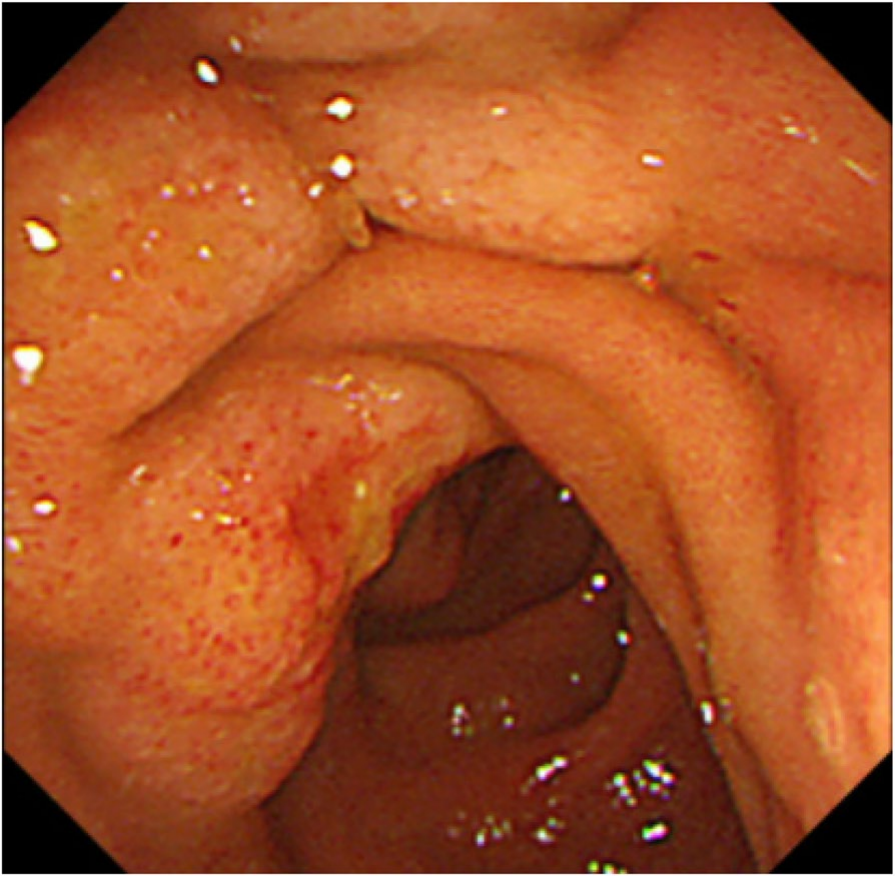
Endoscopic findings of the recurrent tumor with obstructive jaundice. Endoscopy showed poor extension of the descending part of the duodenum and exposure of the recurrent tumor. Biopsy revealed cancer cells with PDA-like histology with scattered signet-ring-like cells

Chemotherapy consisting of mFOLFOX6 plus cetuximab was started in March 2021. CT conducted 8 months after the operation revealed no metastases to lymph nodes or other organs, and the patient was judged to have a complete response (Fig. 5). Chemotherapy was continued until one year after the operation. CT conducted at that time showed no evidence of recurrence; thus, a complete response had been maintained. Chemotherapy was discontinued at the patient’s request. One month later, in March 2022, she was admitted due to obstructive jaundice, with a serum total bilirubin value of 25.04 mg/dL. Serum values of CEA and CA19-9 were 6.2 ng/mL and 30.2 U/mL, respectively. CT revealed choledochal obstruction due to a mass in the pancreas head accompanied by mild dilatation of the main pancreatic duct and dilatation of the intrahepatic bile duct (Fig. 6A). Bulky lymph nodes located in the peri-left adrenal region (Fig. 6B) and swollen lymph nodes in the para-aortic region (Fig. 6C) were observed. Endoscopic retrograde cholangiopancreatography (ERCP) was attempted; however, we failed to confirm the papilla of Vater due to duodenal constriction caused by tumor infiltration and exposure of tumors (Fig. 7). A biopsy was taken from the recurrent tumor of the constricted segment of the duodenum, and PDA with signet-ring cells was diagnosed. Jaundice was improved by percutaneous transhepatic biliary drainage (PTBD); however, cholangitis persisted. Before readministration of chemotherapy, we planned gastrojejunal anastomosis and conversion to internal drainage of the biliary tract in order to resume oral intake and improve cholangitis, and the operation was performed 3 weeks after PTBD, 1 year and 2 months after the prior operation. Exacerbation of the patient’s condition progressed, and 18 days after the second operation, the patient died of the recurrent tumor in April 2022.

With consent from the patient’s family, autopsy was conducted.

## Pathological findings

### Gross findings of the primary ascending colon tumor

**Fig. 8:**
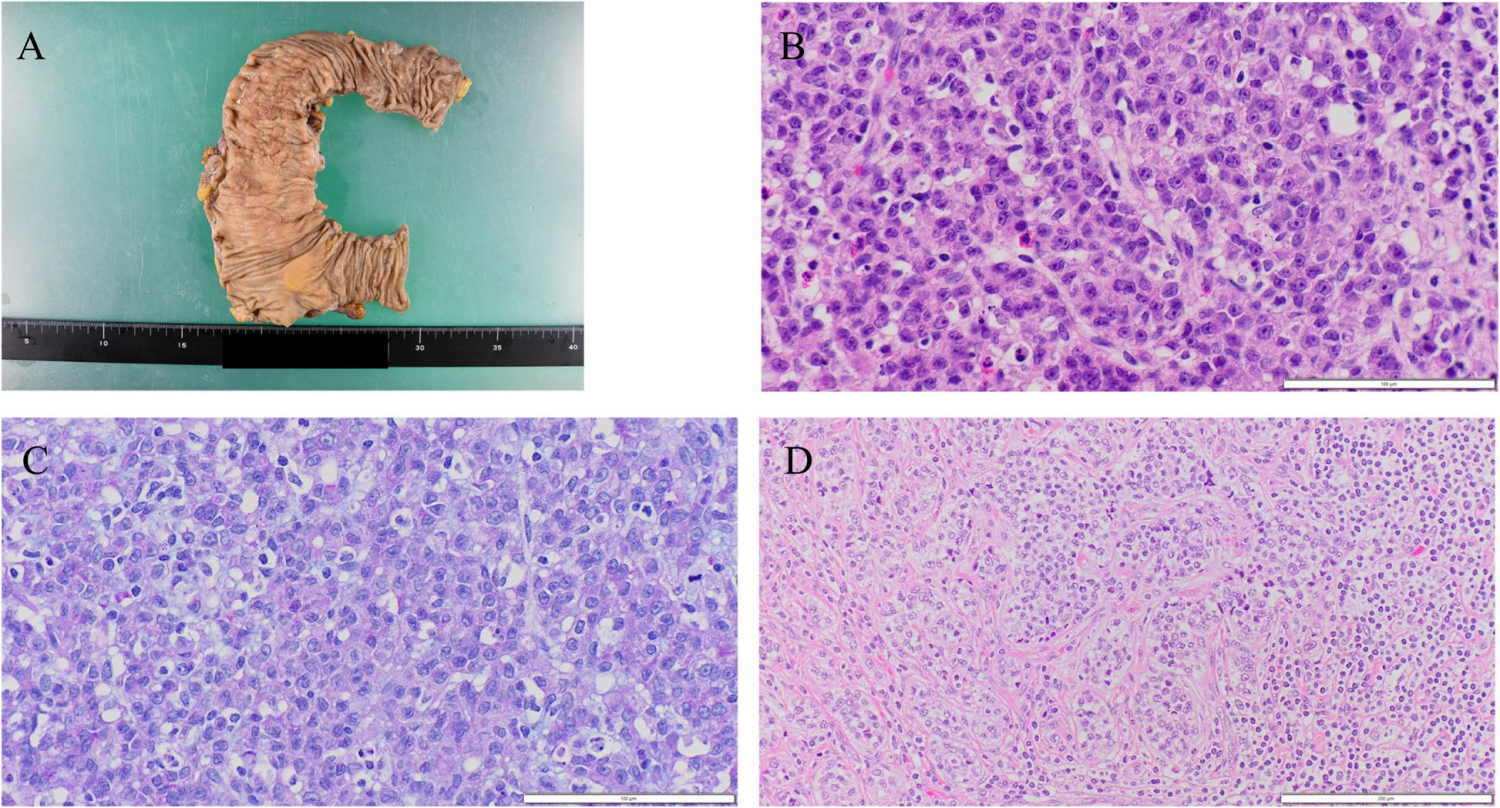
Macroscopic and histopathological features of right hemicolectomy specimens. Gross findings of the ascending colon (**A**), hematoxylin and eosin (HE) staining (**B**) and Alcian blue-periodic acid Schiff (AB-PAS) staining (**C**) of the tumor, and HE staining of the metastatic site of the lymph node (**D**). Accumulation of polypoid gross features of the mucosal surface and wall thickening were found (**A**). The histological type of the tumor is mainly poorly differentiated adenocarcinoma (PDA) (**B**) with scant intracytoplasmic mucin (**C**). Metastatic PDA without a signet-ring cell component is observed in the lymph node (**D**). **B**–**D** Original magnification × 400. Each bar displayed at the bottom right indicates 100 μm

Right hemicolectomy was performed. The ascending colon tumor, which had shown a scirrhous gross appearance on imaging before chemotherapy was administered, was decreased with chemotherapy and showed an unclassifiable macroscopic appearance accompanied by the accumulation of polypoid gross features of the mucosal surface and wall thickening extending the range to 9 cm × 5.5 cm (Fig. 8A). Lymph nodes near the tumor and along the ileocolic artery showed metastases.

### Microscopic findings of the primary ascending colon tumor and metastasized lymph nodes

Five-millimeter-thick serial tissue cross-sections of the full length of the colon cancer, prepared from formalin-fixed and paraffin-embedded tissues, were subjected to pathological examination. The surgical specimens showed extensive fibrosis, with viable tumor cells remaining focally in the mucosa and sporadically in the wall (fibrosis of tumor mass > 50%). Based on the tumor regression grade assigned based on the guidelines of Rödel et al. [15], the effect of preoperative chemotherapy was grade 3. The histological tumor type was mainly consistent with that of PDA, with a small proportion of the moderately differentiated and well-differentiated tubular adenocarcinoma reaching the subserosal layer (Fig. 8B).

Lymphatic invasion was prominent. Extracellular mucin was not observed. Alcian blue-periodic acid Schiff (AB-PAS) staining revealed that most PDA cells had scant intracytoplasmic mucin, although some PDA cells had small amounts of mucin (Fig. 8C). Signet-ring-like features were not identified in any samples, including en bloc resected thickened mesocolon sample. Most parts of the lymph nodes were replaced by fibrous tissue, and the aggregation of macrophages contributed to response to chemotherapy, and minute metastatic clusters consisting of PDAs were revealed in 20 of the 22 resected lymph nodes without signet-ring cell components (Fig. 8D).

Finally, a diagnosis of ypT3N2bM1a, yStage IVA was made [16].

### Histology of the biopsy specimen from the duodenum

**Fig. 9:**
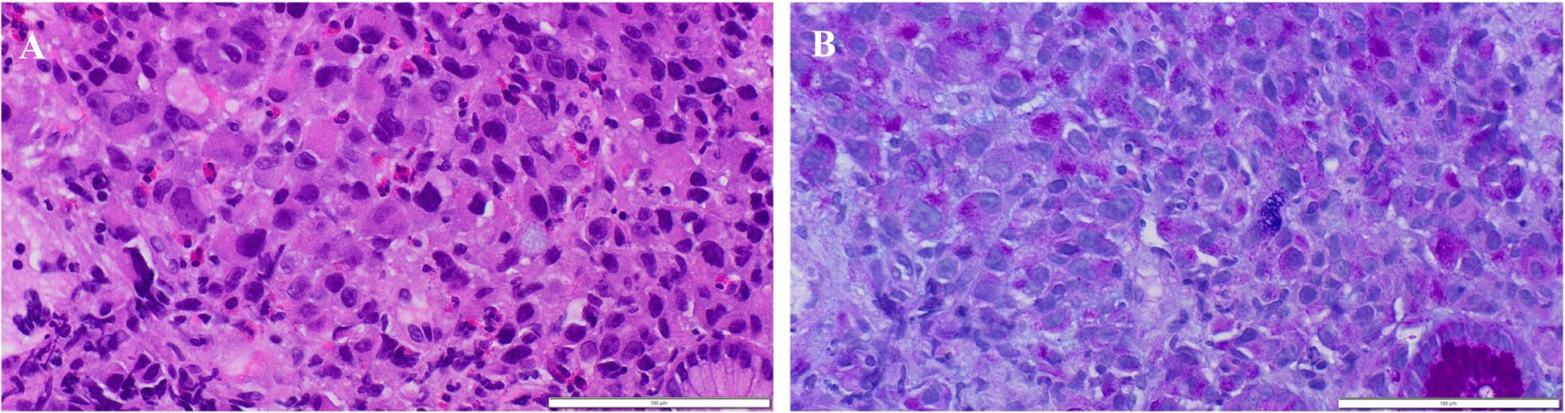
Histopathology of duodenal biopsy specimens. Hematoxylin and eosin staining (**A**) and Alcian blue-periodic acid Schiff (AB-PAS) staining (**B**). Poorly differentiated adenocarcinoma (PDA) with scattered signet-ring cell-like cells is observed (**A**). Signet-ring cell-like cells have enriched intracytoplasmic mucin, and most PDA-like cells also have more intracytoplasmic mucin than the original PDA cells (**B**). **A**, **B** Original magnification × 400. Each bar displayed at the bottom right indicates 100 μm

Hematoxylin and eosin staining showed cancer cells with PDA-like histology with scattered signet-ring-like cells spreading in the lamina propria of the duodenum (Fig. 9A). AB-PAS staining showed enriched cytoplasmic mucin in the signet-ring-like cells (Fig. 9B). In addition, most PDA-like cells also had more intracytoplasmic mucin than the original PDA cells (Fig. 9C).

### Autopsy findings

**Fig. 10:**
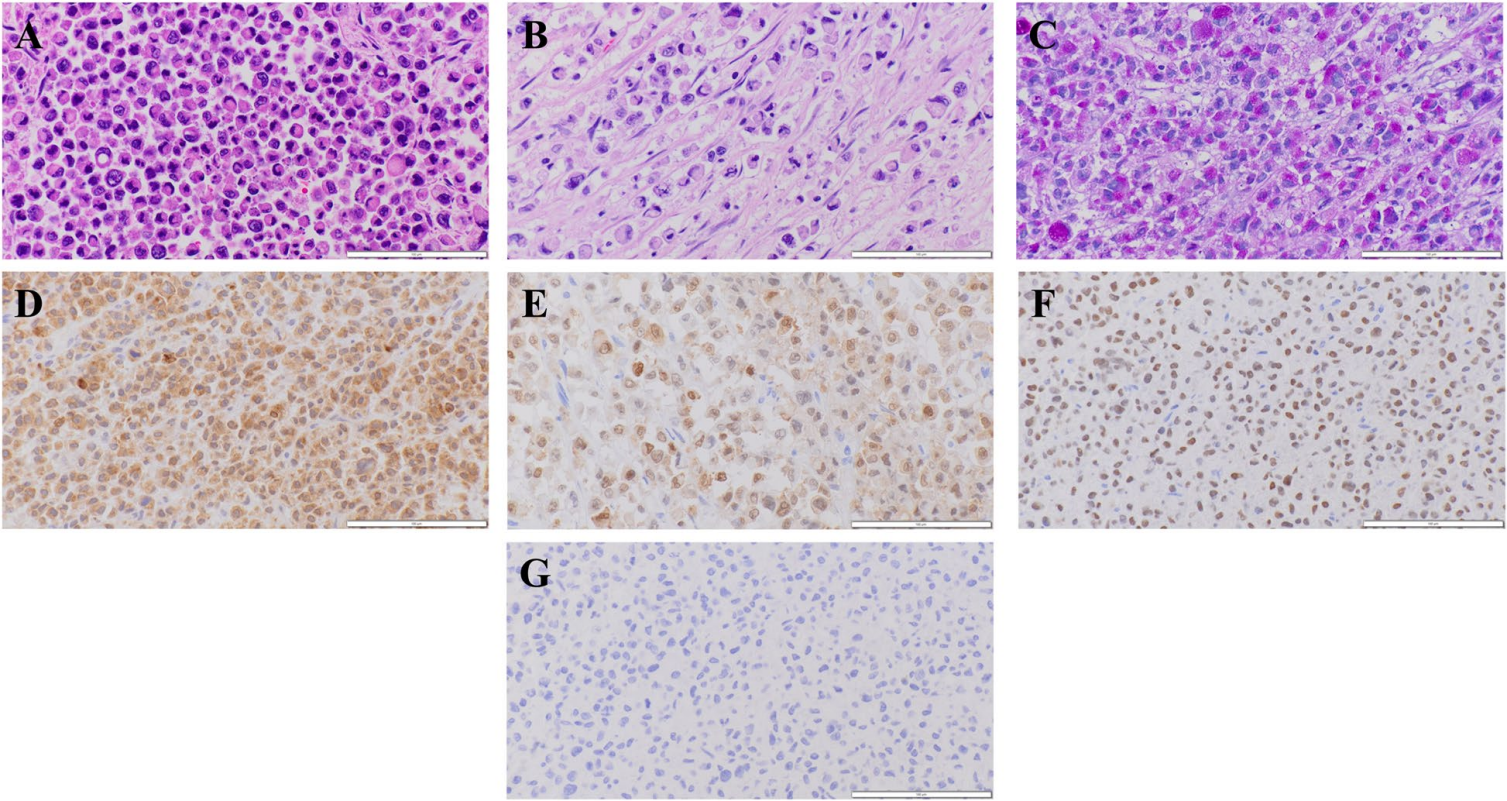
Representative histopathology and immunostaining of autopsy specimens. Hematoxylin and eosin staining (**A** pancreas head, **B** lymph node near left adrenal gland), Alcian blue-periodic acid Schiff (AB-PAS) staining (**C**), and immunostaining for CK20 (**D**), CDX2 (**E**), SATB2 (**F**), and CK7 (**G**). Signet-ring cell-like cells are proliferating and infiltrating (**A** and **B**). These cells have enriched intracytoplasmic mucin (**C**) and are positive for CK20 (**D**), CDX2 (**E**) and SATB2 (**F**) and negative for CK7 (**G**). **A**–**G** Original magnification × 400. Each bar displayed at the bottom right indicates 100 μm

**Fig. 11:**
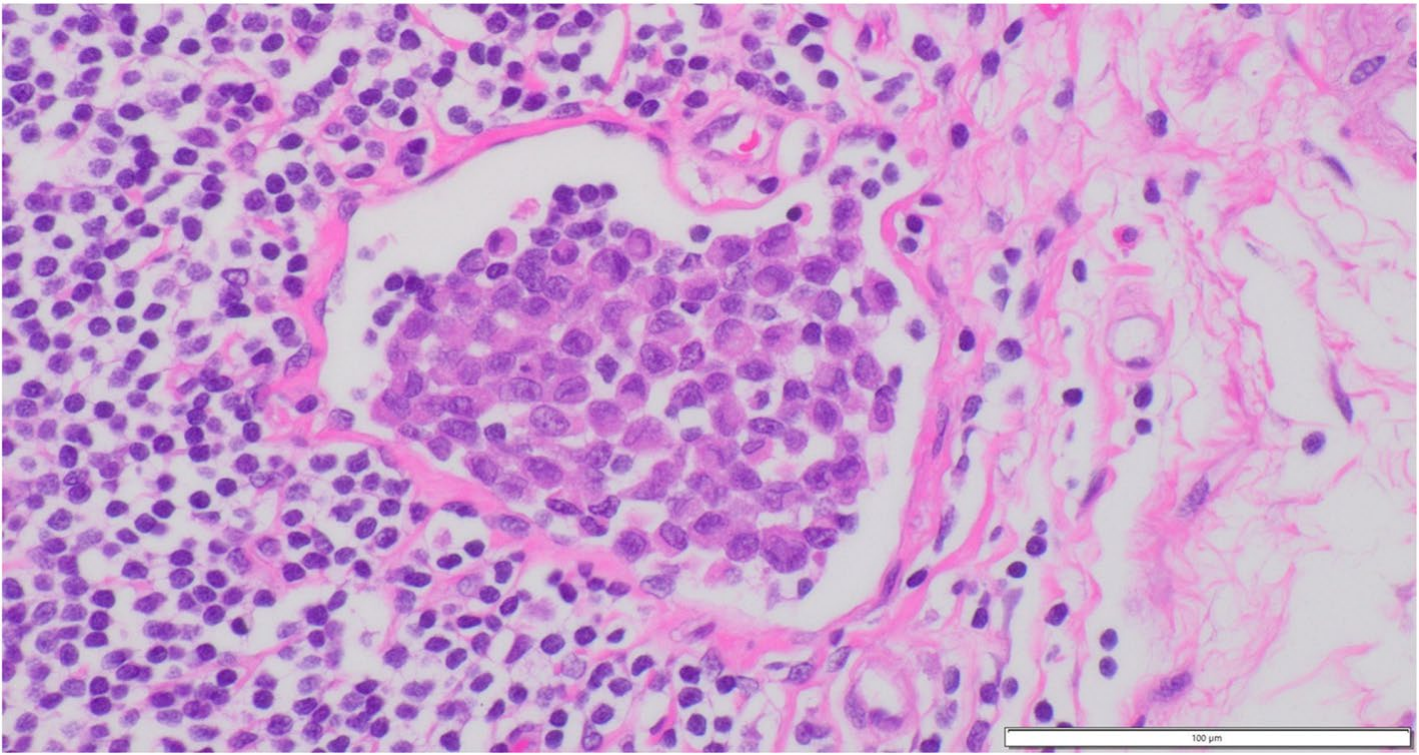
Retrospective evaluation of the right hemicolectomy specimens. Hematoxylin and eosin staining. A very small number of signet-ring-like cells are observed in lymphatic emboli. **A** Original magnification × 400. The bar displayed at the bottom right indicates 100 μm

Autopsy was carried out half a day after the patient’s death and revealed a hard mass located around the pancreatic head (Fig. 10A) accompanied by metastases in the peripancreatic and periduodenal lymph nodes and infiltration into the duodenal wall. Lymph node metastases were also evident in the paraaortic region, mesentery or lesser omentum, hepatoduodenal ligament, peri-left adrenal gland, and left pulmonary hilum. Microscopically, a bulky tumor of the peri-left adrenal gland was revealed to be a metastatic lymph node in contact with the adrenal gland with no metastasis (Fig. 10B). Almost all recurrent tumors consisted of SRCC with a small amount of PDA and slightly moderately differentiated tubular adenocarcinoma components (Fig. 10C). Immunohistochemical staining was carried out and showed positive staining for CK20, CDX2, and SATB2 and negative staining for CK7 (Fig. 10D–G), indicating the recurrent tumor’s primary colon origin. Microsatellite instability was also evaluated by immunohistochemistry staining of mismatch repair (MMR) proteins: mutL homolog 1 (MLH1), postmeiotic segregation increased 2 (PMS2), mutS homolog 2 (MSH2), and mutS homolog 6 (MSH6). An MMR-deficient status was defined as complete loss of nuclear expression of MMR proteins within the tumor. In the tumor cells, nuclear expression of all the MMR proteins was retained. Thus, the present case was considered to be MMR-proficient. Genomic mutation assays for KRAS, NRAS, and BRAF were also carried out using biopsy specimens taken from recurrent duodenal tumors, as autopsy specimens were inadequate for this purpose due to the passage of time from patient death to fixation with formalin; mutation at codon 61 of KRAS was identified despite prechemotherapeutic negativity. We reevaluated the surgically resected specimens to confirm how many of them contained signet-ring-like cells. Only a very small number of signet-ring-like cells were identified in a few lymphatic emboli (Fig. 11).

## Discussion

Despite improvements in OS or PFS of metastatic CRC patients due to advances in chemotherapy, tumors that are unresponsive or develop resistance remain a serious problem. During chemotherapy, alterations in various oncogenes, tumor suppressor genes, and genes involved in the DNA repair process have been detected by liquid biopsy; data from circulating tumor cells, circulating cell-free DNA, exosomes secreted from cancer cells, and epigenetic observations have been analyzed and compared to corresponding data collected prior to chemotherapy [17–19]. It was reported that even in a chemosensitive tumor treated with cetuximab, mutations in oncogenes occurred to some degree [5]. Although such genetic alterations can confer chemoresistance, followed by aggressive biological mechanisms can occur, histological transformation of cancer cells seems to be an extremely rare phenomenon. To the best of our knowledge, there are no reports of histological transformation due to chemotherapy in CRC, whereas a few cases of histological transformation in lung cancer and breast cancer have been reported [6–9]. The present case showed clinically aggressive scirrhous-type ascending colon cancer accompanied by locoregional and distant lymph node metastases at diagnosis and intrinsic chemosensitivity; this case was revealed to mainly consist of PDA but was not associated with extracellular accumulation of mucin or signet-ring cell components, except for a minute cluster in only a few lymphatic emboli in the surgical specimens of ascending colon tumors obtained after mFOLFOX6 plus cetuximab therapy. Chemotherapy plus cetuximab was continued postoperatively, and the tumors had disappeared at 8 months after the operation; this response was maintained for 4 additional months according to CT findings. Discontinuation of chemotherapy plus cetuximab 1 year after the operation resulted in immediate tumor recurrence and rapid expansion. Autopsy specimens revealed that almost all of the recurrent tumors consisted of signet-ring cell histology exhibiting histological transformation. Unlike the insufficient histological confirmation from biopsy specimens from a recurrent site suitable for sampling, the presented patient was autopsied, and many metastasized lesions were investigated; almost all recurrent tumors were replaced by SRCC. Only sporadic reports of histological transformation in lung cancer, such as small cell carcinoma evolving from lung adenocarcinoma, and breast cancer have been reported to be associated with chemotherapy combined with anti-EGFR TKIs, chemotherapy alone, immunotherapy, or hormone replacement therapy [6–9].

Possible mechanisms of histological transformation have been suggested in previous literature, including pluripotent cancer stem cell differentiation and baseline heterogeneity of cancers [20, 21]. Regarding the baseline heterogeneity of the primary cancer, a histopathological diagnosis of PDA of the ascending colon was made, although scirrhous colon cancer showed a characteristic appearance common to SRCC histology of CRCs [22], and a signet-ring cell component was not observed in the primary colon tumor, except for a tiny cluster within a few lymphatic emboli, despite the evaluation of 5 mm slices along the whole length of the tumor. Possibly, the large tumor had a very small amount of SRCC between the sliced specimens, and selective outgrowth of the minute variant component occurred. However, preoperative chemotherapy reduced the primary tumor and metastasized lymph nodes, colectomy was carried out, PDA was proven, and it was not clear whether tiny clusters of SRCC were intrinsic or developed after chemotherapy. After surgical treatment and chemotherapy, biopsy specimens taken from the constricted duodenum also showed a mixture of signet-ring cells among PDA histology. Hematoxylin and eosin staining showed that most metastasized cancer cells had poorly differentiated morphology; however, AB-PAS staining revealed that the signet-ring-like cells had enriched cytoplasmic mucin, and other PDA-like cells also had more intracytoplasmic mucin than the original PDA cells. We observed the process of histological transformation to mature SRCC via immature signet-ring cell-like cells from original PDA cells and ultimately the replacement of PDA cells with signet-ring cells in almost all recurrent tumors.

Among CRCs, SRCC is reported to be diagnosed at a younger age than adenocarcinoma, with a right-sided predilection, large tumor size, scirrhous appearance, aggressive manifestation including a higher frequency of locoregional lymph node involvement, lymphovascular invasion and perineural infiltration, peritoneal dissemination, higher risk of recurrence, and incorrect prognosis [11–14]. Pulmonary and hepatic metastases are uncommon; however, metastases to the brain and bone, including bone marrow, are more common [23]. In this case, the patient was a middle-aged woman with right-sided colon cancer with features similar to those of CRC-SRCC. Given the possibility of an MSI-high status, we investigated MMR protein expression by immunohistochemistry, and the result was MMR-proficient; thus, in this case, MMR did not seem to have an effect on the transformation to SRCC. In accordance with the WHO classification [10], among CRCs, PDA could be categorized as having a signet-ring cell component if present and might have affinity to SRCC; however, the present case scarcely had a component of signet-ring cells and had a histological appearance resembling malignant lymphoma confirmed by negative immunohistochemical staining for CD45. Distinct histological differences existed between these original and recurrent tumors, suggesting that decisive histological transformation occurred. In addition to reports that suggest the existence of an adenoma-SRCC sequence [24, 25], the molecular characteristics of SRCC have been reported to possibly arise from a separate genetic pathway, accompanied by a lower frequency of APC mutations; the presence of less common KRAS, BRAF, and PI3K mutations; frequent SMAD4 mutations; and ordinary MSI-H status [26]. These characteristics are distinct from the frequent alterations in the conventional colorectal adenoma-carcinoma sequence, including APC, KRAS, and PI3K alterations as well as infrequent SMAD4 mutations [12]. The present case showed alteration of the KRAS gene sequence after chemotherapy. Although KRAS mutation does not represent the typical genetic state of SRCC, it suggests that mFOLFOX6 plus cetuximab therapy might promote other oncogene mutations or epigenetic changes, causing the transformation of non-SRCC CRC to SRCC.

Finally, our ability to reveal the mechanisms underlying histological transformation in the presented case was limited because only one recurrent tumor specimen was obtained, which limited the assessment of KRAS, NRAS, and BRAF codon alterations, and additional tumor genomic analysis to identify the unique combination of mutations of various oncogenes or epigenes contributing to transformation to SRCC was not performed. Further accumulation of cases and investigations, including tumor genomic analysis, are necessary to elucidate the mechanisms of the histological transformation of non-SRCC CRC to SRCC.

## Conclusions

We reported an extremely rare case of poorly differentiated scirrhous-type ascending colon cancer that exhibited transformation to SRCC histology after chemotherapy plus cetuximab. This case might suggest that mutations in various oncogenes or epigenes resulting from chemotherapy, especially regimens that include cetuximab, contribute to transforming non-SRCC CRC to SRCC histology and can promote the aggressive clinical progress characteristic of SRCC.

## Data Availability

All data generated or analyzed during this study are included in this published article.

## Abbreviations

EGFR: Epidermal growth factor receptor
TKI: Tyrosine kinase inhibitor
VEGF: Vascular endothelial growth factor
BRAF: V-raf murine sarcoma viral oncogene homolog B1
ICI: Immune checkpoint inhibitor
CRC: Colorectal cancer
OS: Overall survival
PFS: Progression-free survival
DNA: Deoxyribonucleic acid
SRCC: Signet-ring cell carcinoma
PDA: Poorly differentiated adenocarcinoma
CT: Computed tomography
CK AE1/AE3: Cytokeratin AE1/AE3
CDX2: Caudal-type homeobox 2
CD45: Cluster of differentiation 45
KRAS: Kirsten rat sarcoma 2 viral oncogene homolog
NRAS: Neuroblastoma RAS viral oncogene homolog
CEA: Carcinoembryonic antigen
CA19-9: Carbohydrate antigen 19–9
ERCP: Endoscopic retrograde cholangiopancreatography
PTBD: Percutaneous transhepatic biliary drainage
AB-PAS: Alcian blue-periodic acid Schiff
CK20: Cytokeratin 20
SATB2: Special AT-rich sequence-binding protein
CK7: Cytokeratin 7
WHO: World Health Organization
APC: Adenomatous polyposis coli
PI3K: Phosphatidylinositol 3-kinase
SMAD4: Suppressor of mothers against decapentaplegic homolog 4
MSI-H: Microsatellite instability-high
MMR: Mismatch repair
MLH1: MutL homolog 1
PMS2: Postmeiotic segregation increased 2
MSH2: MutS homolog 2
MSH6: MutS homolog 6
